# The DO-KB Knowledgebase: a 20-year journey developing the disease open science ecosystem

**DOI:** 10.1093/nar/gkad1051

**Published:** 2023-11-11

**Authors:** J Allen Baron, Claudia Sanchez-Beato Johnson, Michael A Schor, Dustin Olley, Lance Nickel, Victor Felix, James B Munro, Susan M Bello, Cynthia Bearer, Richard Lichenstein, Katharine Bisordi, Rima Koka, Carol Greene, Lynn M Schriml

**Affiliations:** University of Maryland School of Medicine, Institute for Genome Sciences, Baltimore, MD, USA; University of Maryland School of Medicine, Institute for Genome Sciences, Baltimore, MD, USA; University of Maryland School of Medicine, Institute for Genome Sciences, Baltimore, MD, USA; University of Maryland School of Medicine, Institute for Genome Sciences, Baltimore, MD, USA; University of Maryland School of Medicine, Institute for Genome Sciences, Baltimore, MD, USA; University of Maryland School of Medicine, Institute for Genome Sciences, Baltimore, MD, USA; University of Maryland School of Medicine, Institute for Genome Sciences, Baltimore, MD, USA; Animal and Plant Health Inspection Service, Plant Protection and Quarantine, USDA, USA; Mouse Genome Informatics, The Jackson Laboratory, Bar Harbor, ME, USA; Case Western Reserve University, Cleveland, OH, USA; University of Maryland School of Medicine, Baltimore, MD, USA; University of Maryland School of Medicine, Baltimore, MD, USA; University of Maryland School of Medicine, Baltimore, MD, USA; University of Maryland School of Medicine, Baltimore, MD, USA; University of Maryland School of Medicine, Institute for Genome Sciences, Baltimore, MD, USA

## Abstract

In 2003, the Human Disease Ontology (DO, https://disease-ontology.org/) was established at Northwestern University. In the intervening 20 years, the DO has expanded to become a highly-utilized disease knowledge resource. Serving as the nomenclature and classification standard for human diseases, the DO provides a stable, etiology-based structure integrating mechanistic drivers of human disease. Over the past two decades the DO has grown from a collection of clinical vocabularies, into an expertly curated semantic resource of over 11300 common and rare diseases linking disease concepts through more than 37000 vocabulary cross mappings (v2023-08-08). Here, we introduce the recently launched DO Knowledgebase (DO-KB), which expands the DO’s representation of the diseaseome and enhances the findability, accessibility, interoperability and reusability (FAIR) of disease data through a new SPARQL service and new Faceted Search Interface. The DO-KB is an integrated data system, built upon the DO’s semantic disease knowledge backbone, with resources that expose and connect the DO’s semantic knowledge with disease-related data across Open Linked Data resources. This update includes descriptions of efforts to assess the DO’s global impact and improvements to data quality and content, with emphasis on changes in the last two years.

## Graphical Abstract

**Graphical Abstract:**
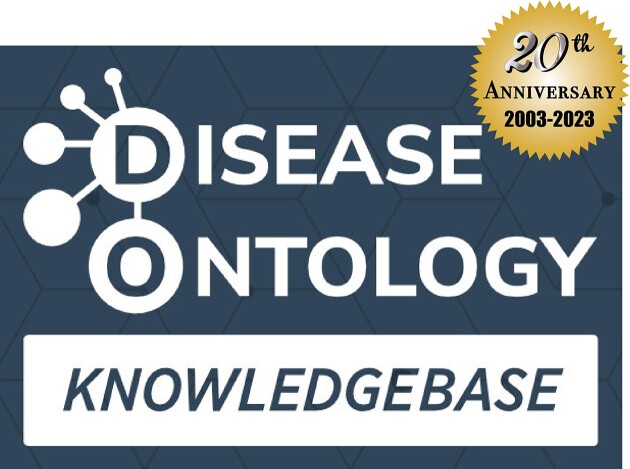

## Introduction

The Human Disease Ontology (DO) (https://disease-ontology.org/) supports an ever expanding disease open-data ecosystem. The DO captures the semantic relationships between diseases and integrates their genetic and environmental mechanistic drivers and characteristics through expanded disease-to-disease linkages. Additionally, the DO maps disease concepts across the breadth of authoritative biomedical resources through >37 000 clinical vocabulary cross mappings. Along with continuous expansion of disease terms, definitions and synonyms, the DO has become a cornerstone, community resource serving hundreds of biomedical, clinical, ontology and software resources.

To enhance curation quality and speed the DO has integrated rigorous quality control protocols, including establishing and adhering to formal data design patterns, utilizing continuous integration testing, and shifting quality control (QC) and previously manual curation tasks to semi-automated, data driven, ML-empowered processes. These behind the scene advances include advancement of the DO’s semi-automated workflow to vet and assess data prior to integration, to produce ML-ready datasets with each monthly data release, to run quarterly data validation tests, to identify DO resource usage and to determine the geographic distribution of the DO’s user community.

Here, we report on advances since the DO’s previous Nucleic Acids Research Database issue publication (NARdb) (1), highlighting the expansion of the DO from a genomic resource into a knowledgebase (DO-KB), providing novel tools that interconnect biomedical knowledge across scales from disease features to differential diagnosis. This update also marks the DO’s 20th anniversary, highlighting the evolution of the DO resource to meet the evolving needs of an ever expanding disease knowledge corpus and user community.

## The DO's 20-year journey

2023 marks the 20th year of the DO project. The idea of establishing a semantically structured ontological representation of human diseases began as an idea sketched out by Rex Chisholm and Warren Kibbe on the back of a napkin on the way home from a Gene Ontology meeting. The inspiration was born by the need in the biomedical community to build an ontology representing a semantic classification of human diseases. The DO project formalized with a first release in 2003, followed by incremental expansion of the DO team, and the establishment of an etiology-based disease classification. The DO expanded in the next decade to serve a growing user community and, in the process, become a globally utilized community resource. The DO initially integrated disease terms in the OBO format, shifting to an enhanced disease data Web Ontology Language (OWL) (https://www.w3.org/OWL/) representation to enable the representation of direct and indirect parent relationships. Defining features of diseases, the DO began to integrate disease axioms which provided disease classification from alternative points of view, e.g. anatomical view or cell of origin. Moving from a Creative Commons CC BY 4.0 to a CC0 1.0 license (https://creativecommons.org) enhanced the DO’s utility to pharma and clinical resources. By making the ontology freely available for any use, the DO chose to enable broad utilization, resulting in an enhancement of the DO usage for machine learning (ML) and artificial intelligence (AI) applications. Today, the DO has expanded our disease feature integration to include 15 other OBO Foundry (http://obofoundry.org/) ontology imports, most recently adding age of onset. Starting from a few thousand disease terms from MeSH (Medical Subject Headings) (http://www.ncbi.nlm.nih.gov/mesh/), ICD-9 (WHO’s International Classification of Diseases) (http://icd9cm.chrisendres.com/) and NCI Thesaurus (https://ncithesaurus.nci.nih.gov/ncitbrowser/) organized in an alphabetical list, the DO has evolved into a hierarchically structured resource that continually integrates community-derived disease classifications, adding on average 500–700 new disease terms each year. The DO standardizes the characterization of disease information, providing consistent data representation through definition design patterns, with DO’s OBO and OWL trees providing semantic search capabilities for querying the disease classification. The DO ingests and semantically represents expertly curated disease knowledge.

## Disease ontology knowledgebase (DO-KB)

The DO-KB has been established to enhance data discovery, by exposing the semantic relationships encoded in the DO’s and other Linked Open Data (LOD) knowledge graphs (https://www.w3.org/wiki/SweoIG/TaskForces/CommunityProjects/LinkingOpenData) through an integrated data system. For example, with the DO-KB tools, users can answer the following questions:

(DO-KB SPARQL service)

- How many DO disease terms include cross-references to MeSH?
- What are all of the ‘metabolic diseases’ in the DO?
- What is the extent of cross-reference connections between MeSH and OMIM?
- Which proteoforms from the PRO (Protein Ontology) are associated with progression in cancer?

(DO-KB faceted search interface)

- Which diseases are associated with a loss of function variant?
- Which autosomal dominant inheritance diseases are associated with a pediatric age of onset?
- What are the diseases that involve inflammation and duplication of a chromosomal region?
- Which diseases are characterized by polyuria resulting from an autosomal recessively inherited gene mutation?
- What are the diseases that involve the lower leg (lower limb segment) and are characterized by the phenotype ‘Abnormal skeletal morphology’?

### Launching the DO-KB disease open science ecosystem

In June 2023, the revamped DO project website was released, with a new look and feel, retooling the organization of resource pages, creating additional pages for the DO-KB and DO-KB tools, along with additional tutorial resources and statistics representing the global reach of the DO user base. The top sections (drop down menus) of the website now include: Disease Ontology, DO-KB, About, Community, Tools & Resources, Outreach, Curation and Downloads. The new landing page provides access to the previous home page, with the DO OWL and OBO tree views (‘Disease Ontology’: https://disease-ontology.org/do), side-by-side with the new DO-KB resources. The DO-KB focuses on knowledge generation through the development and implementation of efficient processes to collect, collate, organize, annotate, disseminate, and connect disease-related data.

**Figure 1.**
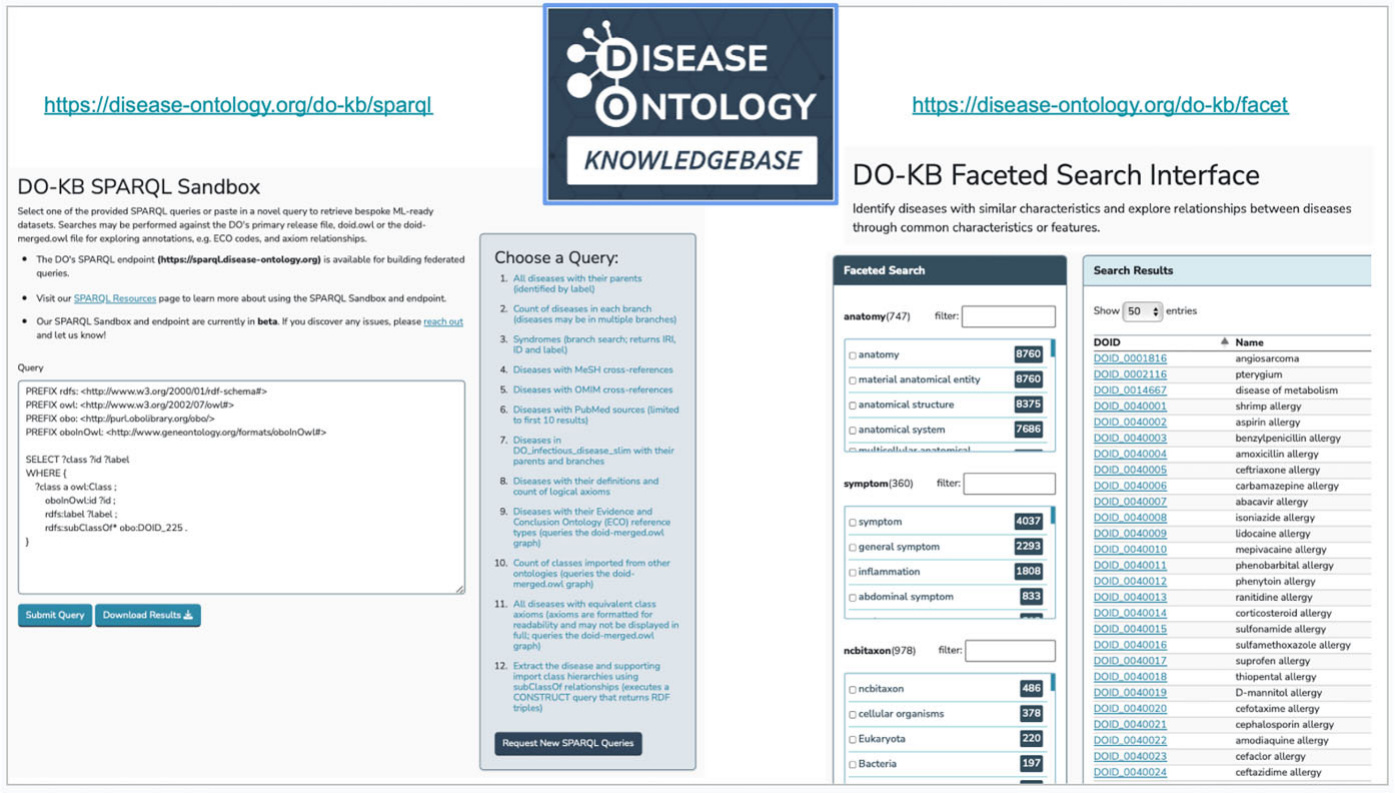
The DO-KB SPARQL Sandbox and Faceted Search Interface tools.

Taking the connection of human disease data to an aspirational level, the DO team envisioned a disease open science ecosystem that would enable the identification and exploration of disease related data across resources. Towards this idea, we developed the DO-KB (https://disease-ontology.org/do-kb/) and the new data exploration DO-KB tools: the DO-KB SPARQL service, comprised of a DO-KB SPARQL endpoint and an online ‘sandbox’; and the DO-KB Faceted Search Interface (Figure 1). These tools offer new disease data exploration capabilities through SPARQL federated queries and connected disease data mining. The DO-KB SPARQL service remotely executes database queries and data retrieval from other Open Linked Data (https://www.w3.org/wiki/SweoIG/TaskForces/CommunityProjects/LinkingOpenData) resources. DO-KB provides attribution using the resource link in the SPARQL query, (e.g. for the Protein Ontology, PREFIX paf: <http://pir.georgetown.edu/pro/paf#>) and by including the ontology URI in the retrieved dataset (e.g. http://purl.obolibrary.org/obo/PR_000000053). Thus the DO-KB is in compliance with CC-BY licensing of these resources (https://creativecommons.org/share-your-work/cclicenses/). The onus is on the data user to be aware of and comply with the source data licensing. Advancing the DO’s support of an open disease ecosystem, the DO-KB makes disease data across biomedical data resources more FAIR (Findable, Accessible, Interoperable, and Reusable). Our goal for this effort was to facilitate exploration of disease mechanisms and drivers, to enable discernment of disease-to-disease connections, and to make the results of queries available as downloadable, ML-ready datasets.

### DO website expansion

The Tools & Resources and Outreach dropdown menus guide users to explore related resources (https://disease-ontology.org/resources/related-resources) including the DO’s Symptom Ontology and Pathogen Transmission Ontology, DO.utils and DO-KB tutorials (https://disease-ontology.org/outreach/tutorial/). The expanding list of resources using the DO has been moved to the new Community > Use Cases page (https://disease-ontology.org/community/use-cases), where resources are grouped into three categories: Ontologies, Resources: a grouping of non-ontology tools that are web accessible, and Methodologies, which include statistical algorithms and AI/ML approaches that utilize the DO. The Curation page (https://disease-ontology.org/curation/) now includes links to external websites of medical vocabularies frequently referenced by the DO team and a link to documentation displaying the most common logical axiom patterns used in the DO. Direct navigation to disease term pages on disease-ontology.org has not changed (e.g. https://www.disease-ontology.org/?id=DOID:4) and as of May 2022 the persistent URLs (PURLs) of each disease term redirect to these pages instead of disease term pages maintained by Ontobee (2). The DO disease term pages hosted at disease-ontology.org provide the most up to date resource of information. The DO terms are also provided on disease pages hosted by Ontobee (https://ontobee.org/ontology/DOID), Bioportal (https://bioportal.bioontology.org/ontologies/DOID), and the European Bioinformatics Institute's Ontology Lookup Service, OLS4 (https://www.ebi.ac.uk/ols4).

### DO-KB tools: SPARQL Sandbox and Endpoint

The DO-KB SPARQL service provides access, via the ‘SPARQL Protocol and RDF Query Language’ (SPARQL, v1.1) (https://www.w3.org/TR/sparql11-overview/), to explore DO diseases, disease features, cross references and disease mechanisms. The service enables users to access, connect, subset, combine and reorganize disease-related data from other Resource Description Framework (RDF) formatted content across the Web with federated queries. The service can be accessed from the DO-KB’s SPARQL Sandbox (https://disease-ontology.org/do-kb/sparql), an HTML-based query editor designed to enable quick, iterative exploration and in browser queries, or programmatically through its SPARQL endpoint (https://sparql.disease-ontology.org/). By default, searches will be executed against the DO’s primary release file, doid.owl. The doid-merged.owl file is also available for querying by including a GRAPH statement, to query data from disease-related terms imported from other ontologies, e.g. evidence codes (3) or symptoms. The DO-KB SPARQL Sandbox supports SELECT queries, with tabular results displayed in the browser and downloadable in .csv format and CONSTRUCT queries, which return graph data as a triple store (.ttl). A suite of editable queries are included in the Sandbox. The initial set of queries will be updated periodically. Bespoke queries can be requested for inclusion in the query set. The initial set of 15 queries demonstrate how to obtain disease identifiers, labels or cross references, how to query the doid-merged.owl file and how to execute federated queries to retrieve disease-related data on the web.

The DO-KB SPARQL endpoint (https://sparql.disease-ontology.org/) supports federated queries and HTTP-based requests. In addition to SELECT and CONSTRUCT queries, the endpoint supports ASK and DESCRIBE queries. Additional output serializations are also available via the endpoint including all those supported by Apache Jena (https://jena.apache.org/), such as JSON, csv, and xml for SELECT and similar queries and RDF/XML, JSON-LD and n-triples for CONSTRUCT queries.

### DO-KB tools: Faceted Search Interface

**Figure 2.**
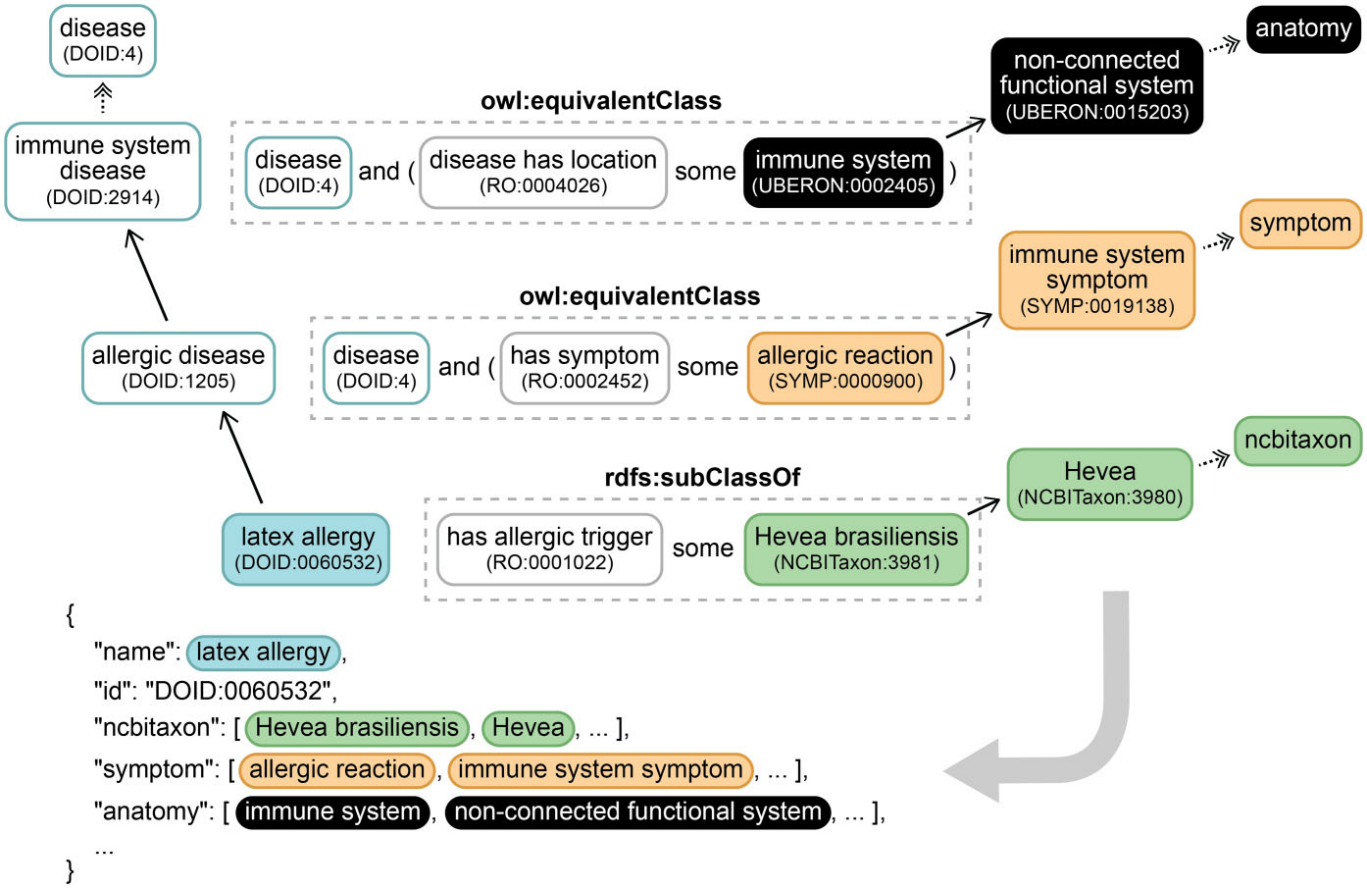
The DO-KB ‘OWL Flattener’ prepares data, from the doid-merged.owl file, for search with DO-KB Faceted Search Interface. An example with the disease ‘latex allergy’: (light blue) is shown. The OWL Flattener traverses up the rdfs:subClassOf relationships (black arrows) of the disease hierarchy extracting all imported terms from logical relationships (dashed outlines). It also traverses up the hierarchies of imported ontologies (anatomy: black; symptom; orange, and ncbitaxon; green) to the root node (multi-node traversal represented by black dashed arrows). All imported terms and ancestors identified are ‘flattened’ into a JSON key-value list representation for the disease (bottom of figure), essentially flattening hierarchically represented relationships. Only asserted rdfs:subClassOf relationships are shown but inferred relationships are also traversed.

The DO-KB Faceted Search Interface provides a new approach to identify the connectivity between diseases and to retrieve ML-ready disease datasets by exploring the diseaseome as a network of anatomic sites, cells of origin, age of onset, phenotypes, symptoms, variant type and age of onset, along with genetic and environmental drivers. The breadth of disease facets will be expanded as the DO integrates additional ontologies to describe features of complex diseases including factors driving social determinants of health (4). The DO defines characteristics and features of diseases through OWL logical statements. These statements include one or more terms from the OBO Relations Ontology (RO) (http://obofoundry.org/ontology/ro.html) that define the type of disease-to-feature relationship(s). As illustrated in the DO’s representation of the disease ‘latex allergy’ (DOID:0060532), which includes the RO term ‘has allergic trigger’ (RO:0001022) and the imported term ‘Hevea brasiliensis’ (NCBITaxon:3981), essentially stating that the disease is triggered by a reaction to the Brazilian rubber tree (Figure 2). An important aspect of OWL is its logical relationship inheritance, where each disease inherits the relationships of its parent(s), grandparent(s) on up the disease hierarchy. Latex allergy, therefore, inherits the relationship to ‘allergic reaction’ (SYMP:0000900) from its parent ‘allergic disease’ (DOID:1205). These logical statements, also referred to as logical axioms, enable automated reasoning to expand the classification of diseases and support powerful semantic queries. While data represented in this format offers a rich environment for defining and exploring data relationships, exploration of this encoded information has proven to be technically challenging, requiring specialized knowledge of programs dependent on OWL reasoners, such as the OWL API (http://owlcs.github.io/owlapi/), to access. The DO-KB Faceted Search Interface has been designed specifically to break down those barriers and open wide the doors to the disease data contained in the DO.

**Figure 3.**
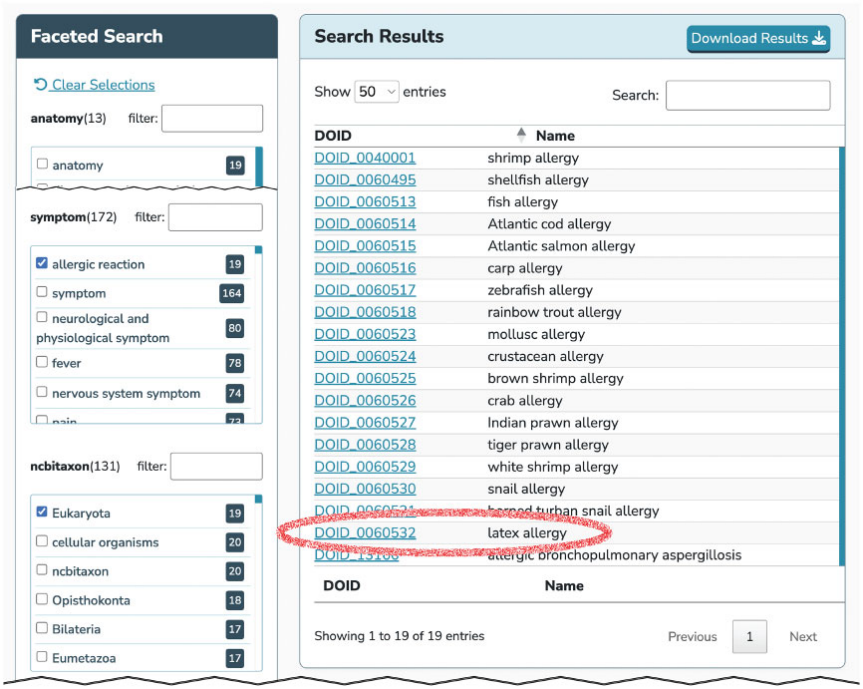
The DO-KB Faceted Search Interface enables discovery of disease-to-disease connections. Latex allergy, caused by allergic reaction to the tree *Hevea brasiliensis*, is among search results for diseases with the ‘allergic reaction’ symptom related to organisms in the kingdom ‘Eukaryota’.

The DO-KB Faceted Search Interface currently includes 12 facets. Each facet identifies the number of diseases associated with the terms in each facet (import). For example, there are 8782 diseases associated with an anatomy term and 2874 disease terms associated with the anatomy term ‘nervous system’. Each facet term acts as a filter showing only those diseases related to the terms selected. Direct and inherited ontology terms can be selected to identify diseases with similar characteristics or features. For example, ‘allergic reaction’ (SYMP:0000900) could be selected from the symptom facet and ‘Eukaryota’ (NCBITaxon:2759) from the ncbitaxon facet in order to identify all allergic diseases caused by eukaryotic organisms (Figure 3). Terms selected within a facet are treated as Boolean OR searches, e.g. selecting ‘allergic reaction’ (SYMP:0000900) and ‘fever’ (SYMP:0000613) within the symptom facet will retrieve diseases with either of those symptoms. Terms in different facets are treated as Boolean AND searches, e.g. lung (anatomy; UBERON:0002048) and epithelial cell (cell type; CL:0000066) will retrieve diseases located in the lung derived from epithelial cells. As terms are selected in a facet, the counts and terms of other facets dynamically update to include only those that are related to the current filtered set of diseases. Filtered disease list results can be downloaded as .csv files.

The advanced query option of the DO-KB Faceted Search Interface provides additional search flexibility for searching based on relationships between terms or groups of terms. The online Faceted Search Interface tutorial (https://disease-ontology.org/outreach/tutorial/) provides details on how to design advanced queries.

The DO-KB Faceted Search Interface is powered by Elasticsearch (https://www.elastic.co/elasticsearch/). This back-end service was chosen to optimize processing speed and enable advanced programmatic logical queries. To format the data for Elasticsearch, a new ‘OWL Flattener’ program was developed to extract terms and their associated logical statements from the DO OWL files, to arrange the DO terms in JSON key-value lists and to organize those terms into dynamic facets using Java and the Java OWL API package. The DO-KB ‘OWL Flattener’ program converts the hierarchical DO OWL file into a flat list of documents. For each disease in the DO, the OWL Flattener extracts the import terms from direct logical axioms along with their parent terms, identified by following the hierarchical rdfs:subClassOf relationships up to a root node. The OWL Flattener then walks up the disease hierarchy pulling down all identified terms in the same manner for each parent term and ‘flattening’ these to the initial disease. The program caches the identified term relationships to improve processing, using memoization, in order to avoid the expensive operation of reidentifying relationships that have already been determined. In the resulting JSON key-value list for each disease, the keys correspond to the import and the set of terms as values (Figure 2).

The OWL Flattener Java code has been made available under CC0 licensing as a repository in the Disease Ontology GitHub organization (https://github.com/DiseaseOntology/OWLFlattener). The OWL Flattener has been modified to enable it to be used on any ontology project, providing the utility to flatten the hierarchical axioms down from ancestors to children. The Flattener has been revised to address its root node dependency, to accept the term ID as a command line input, so that it could be repurposed by other projects. For the DO-KB the flattener logic has a hardcoded string which is the DOID root node, that is used as part of the flattening to know when the process has gone too far up the tree. The flattener cuts off the axiom imports one level beneath the root node so the root node needs to be indicated.

### DO-KB infrastructure

**Figure 4.**
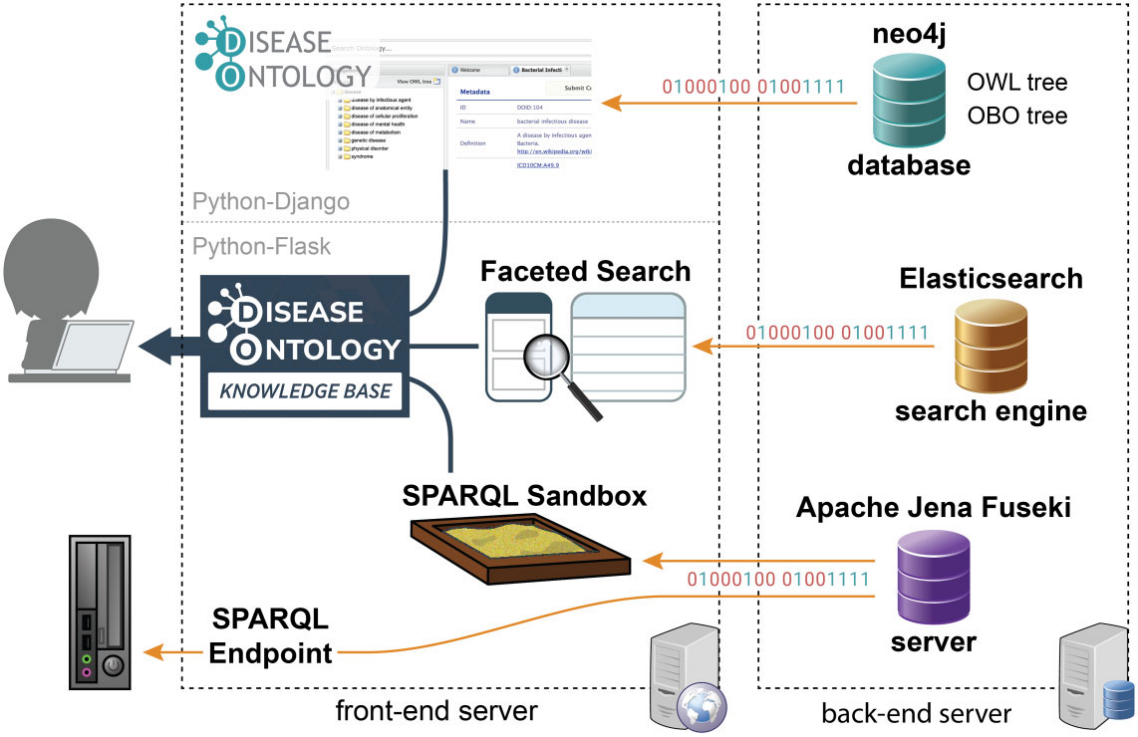
The updated infrastructure supporting the DO-KB. The Apache web front-end server integrates the original disease-ontology.org site (using a Python-Django web app) with the new DO-KB pages powered by a Python-Flask framework, including pages for the DO-KB SPARQL Sandbox and Faceted Search Interface, and seamlessly serves these pages to users of disease-ontology.org (dark blue lines). The back-end server provides data and search capabilities (orange lines) to the front-end pages and the SPARQL endpoint using the three depicted services. The original hierarchical trees, search, and term pages of disease-ontology.org are powered by a neo4j database (light blue database icon), the new Elasticsearch search engine (orange database icon) serves data to the DO-KB Faceted Search Interface and an Apache Jena Fuseki server (purple database icon) powers the SPARQL Sandbox and Endpoint.

Both the DO front- and back-end servers were augmented to launch the DO-KB resources (Figure 4). The front-end server continues to serve up the original web pages of disease-ontology.org using a Python-Django (https://www.djangoproject.com/) powered web application, while the new DO-KB pages are driven by a lighter-weight Python-Flask framework (https://flask.palletsprojects.com). These apps are integrated and served to users using an Apache web server. The OBO tree, OWL tree, and disease term pages are still powered on the back-end server by a neo4j database (https://neo4j.com/), while an Elasticsearch search engine was added to power the new DO-KB Faceted Search Interface and an Apache Jena Fuseki server (https://jena.apache.org/documentation/fuseki2/) was added to power the DO-KB SPARQL Sandbox and Endpoint. The SPARQL endpoint on the front-end server, situated at https://sparql.disease-ontology.org/, acts as a passthrough to the Apache Jena Fuseki server for programmatic access when requests include a query parameter. To prevent confusion, requests that do not contain a query are redirected to the SPARQL Sandbox.

## Content expansion

In the DO’s previous NARdb publication, we described the 100th Github release of the Disease Ontology (v2021-08-17) which included 10862 disease terms with 76% (8, 312) defined with textual definitions. Since that publication, the DO (v2023-08-08) has expanded to include 11367 diseases with 79% (8996) including textual definitions. Additionally, >2400 DO diseases have been updated to reflect the current state of biomedical research. The DO actively engages the clinical community regarding content integration. Clinicians and medical students from the University of Maryland School of Medicine and Case Western, as members of the DO team since 2018 (https://disease-ontology.org/about/do-team), contribute substantially to the DO’s content. For example, in the past two years, the DO Clinical team has revised the classifications of diabetes and asthma as part of our Complex Disease Modeling effort (4). The DO’s content has benefited from the ongoing review efforts by numerous clinician researchers, including collaborators from CIViC and ClinGen (Pediatric Cancer Taskforce), focusing on pediatric cancer data representations, age of onset determinations and the integration of ICD-O (Oncology) identifiers and nomenclature revisions. Disease terms have been added and updated across all branches of the DO’s disease hierarchy. Targeted review and updates include rare diseases (65 new, 841 updated) and diseases involving digenic inheritance (32 new). Curation effort has focused on the integration of revised disease classifications for hereditary spastic paraplegias, autosomal recessive and dominant intellectual developmental disorders, developmental and epileptic encephalopathies, hypomyelinating leukodystrophies, lymphomas, sexual dysfunction, eye diseases and syndromes, among others. A complete list of diseases added to the DO in the past two years is available as a ML-ready dataset in the DO’s GitHub repository (https://github.com/DiseaseOntology/HumanDiseaseOntology/blob/main/DOreports/new_diseases_2021-23.tsv). The details of the revised classifications can be gleaned from the DO’s monthly release notes (https://github.com/DiseaseOntology/HumanDiseaseOntology/releases).

The DO continues to enhance interoperability, acting as a bridge to and between other clinical vocabularies. Through the DO’s ongoing collaboration with CIViC (5) and ClinGen (6), >450 cross references to the International Classification of Diseases for Oncology (ICD-O) (https://www.who.int/standards/classifications/other-classifications/international-classification-of-diseases-for-oncology) have been reviewed and added to the DO. In addition, the DO’s curation pipeline has been revised to include automated matching assessments between diseases in the DO and other resources, to identify and rank potential matches based on identifier, disease name and synonyms. Utilizing this new system, we have reviewed and revised DO mappings to the Online Mendelian Inheritance in Man (OMIM) (7), Genetic and Rare Diseases Information Center (GARD) (8), Orphanet (9), and MeSH, resulting in the addition of 1100 cross references, bringing the total cross references contained in the DO to over 37 000. Working with partners, such as biomappings (10) for MeSH comparisons, disease cross reference representations were reviewed and improved and previously time intensive curation tasks were automated.

The DO publishes subsets (‘slims’), updated with each release, to simplify and tailor its use by collaborators, consortia, and individual researchers. Two new subsets have been added. The DO_CFDE_slim was created to support the Common Fund Data Ecosystem (CFDE) (11) in its effort to align and harmonize disease-related information across data produced by 11 National Institutes of Health (NIH) Common Fund Programs’ Data Coordination Centers. The CFDE slim includes DO’s upper-level disease classes except for the ‘disease of anatomical entity’ and ‘disease of cellular proliferation’ branches which have been split to directly include major anatomical entities, ‘cancer’ and ‘benign neoplasm’. For many years various infectious disease subsets have been produced through manual curation to support specific communities. The new DO_infectious_disease_slim, which includes all infectious diseases, is now produced programmatically to support broader analysis by the infectious disease community.

### Symptom Ontology updated

In the past 2 years, the Symptom Ontology (SYMP) content has been extensively reviewed and revised. The current Symptom Ontology release includes 894 classes (with 65 new SYMP terms added since the previous DO NARdb paper). This work involved the addition of logical axioms linking diseases and symptoms in the DO (https://disease-ontology.org/resources/symptom-ontology) resulting in an expansion of our disease-to-symptom defined relationships to include 4055 diseases associated with a SYMP term, as reported in the DO-KB Faceted Search Interface. Revisions of the Symptom Ontology, in detail, are available in GitHub (https://github.com/DiseaseOntology/SymptomOntology). This work focused on the review and revision of the hierarchy of each branch and the addition of definitions, reformatting of current definitions for consistency, the addition of database cross references and synonyms (exact & related). Exact synonyms (duplicate_label_synonym issues) that were identical to the term in question were removed. This work resolved additional duplicate_label_synonym issues, merged colloquial terms and added synonyms. The Symptom Ontology release process was updated to an automated release and QC system in May 2022.

### SPARQL queries & ML-ready datasets

ML-ready reports are generated during each DO release with bespoke reports devised upon request (https://github.com/DiseaseOntology/HumanDiseaseOntology/tree/main/DOreports). The SPARQL queries utilized to build these reports are also available in DO’s GitHub (https://github.com/DiseaseOntology/HumanDiseaseOntology/tree/main/src/sparql/DOreports). These DOreport (tab-separated value (.tsv) formatted) datasets improve data accessibility for researchers and make data ready for ML applications, reducing the need for data transformation. Exported data includes the identifiers, labels and asserted superclasses for all diseases in the DO, cross references, and the complex logical equivalent class and subclass axioms formatted as text. Additional user-requested, one-time reports are also available in the DOreports GitHub folder.

### Quality control and release automation

Increasing the use of automation has greatly enhanced the DO’s capacity for assessing and integrating data. Expanding the suite of bespoke SPARQL queries (https://github.com/DiseaseOntology/HumanDiseaseOntology/tree/main/src/sparql), has hardened the DO’s build assessment, enhancing the verification of ontological rigor. These queries assess the structure and format of IDs and alternate IDs, evidence and conclusion codes, cross references, and logical axioms using phenotypes, symptoms, and diseases as features. Automating the DO’s quarterly, automated checks now includes the assessment of URL validity, identifying variations in the DO’s definition formats, looking for spelling errors or inconsistencies within all text of the ontology. Automated identification of new disease terms from the clinical vocabularies of GARD, Orphanet and NORD (National Organization for Rare Disorders) database (https://rarediseases.org/rare-diseases/) informs and enhances the DO’s ongoing data curation efforts. Additionally, the DO_rare_slim is now automatically populated by adding DO terms that include cross-references to authoritative rare disease resources (GARD, NORD or Orphanet).

## Global impact and outreach

### Identifying DO usage

**Figure 5.**
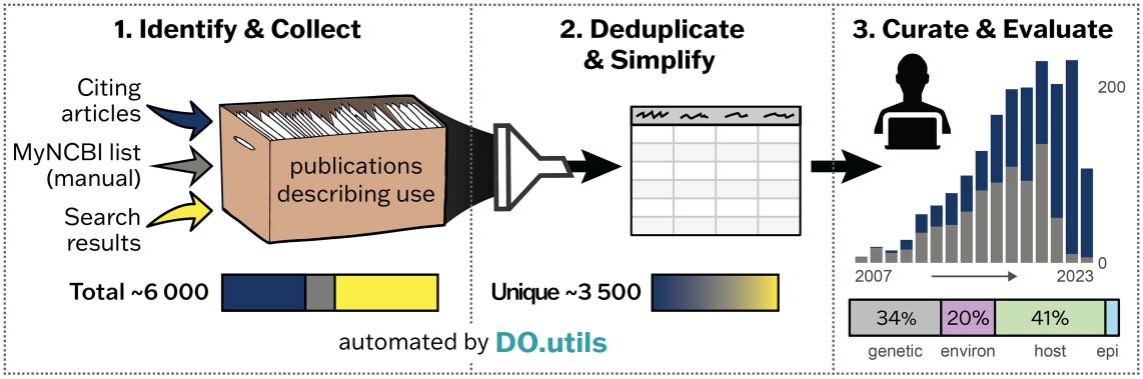
The semi-automated ‘Assessing Resource Use’ workflow utilized to more fully capture resource usage, consists of three major steps. The first two steps of the workflow, automated with the R package DO.utils includes the identification and collection of scientific publications (section 1) from citing literature (dark blue arrow & boxes), manual identification (gray arrow & boxes) and tailored searches (yellow arrow & boxes), and the deduplication and simplification of these records into an easy to view tabular format (section 2). The last step (section 3) is curation and evaluation of these publication records. Results of applying this workflow for the DO are shown as proportional colored boxes with total numbers. Section 3 shows the result of curation and evaluation with a histogram displaying the number of scientific publications that cited or used the DO each year from 2007 to July 2023 identified by our prior manual approach (grey) versus the total from use of this workflow (dark blue), excluding those identified by tailored searches (yellow - Section 1), and the proportion of publications using the DO over the last year binned by etiological research (genetic, environmental, host, and epigenetic factors).

Assessing resource use is an ongoing challenge for biomedical resource providers. Manually intensive efforts, to assess resource use from scientific literature, were replaced with a new, semi-automated workflow that has expanded our awareness of the number and types of uses (Figure 5). The DO.utils R package (12) (https://github.com/DiseaseOntology/DO.utils) powers automation within the workflow and was developed to be amenable for implementation by any biomedical resource. This work has identified the number of biomedical resources published between 2008 and July 2023 that use the Disease Ontology and grouped them by sub-continent. The DO and DO-KB usage metrics are updated quarterly. These resources are identified primarily from publications citing the DO, tallied by country of origin and then grouped by sub-continent, with resources created by researchers in more than one sub-continent included in the count of each. The list of individual biomedical resources can be found on the DO’s Use Cases (https://disease-ontology.org/community/use-cases) page. A tutorial describing how to use the DO.utils resource is available at: (https://diseaseontology.github.io/DO.utils/articles/obtain_use_records.html).

### Measuring success

**Figure 6.**
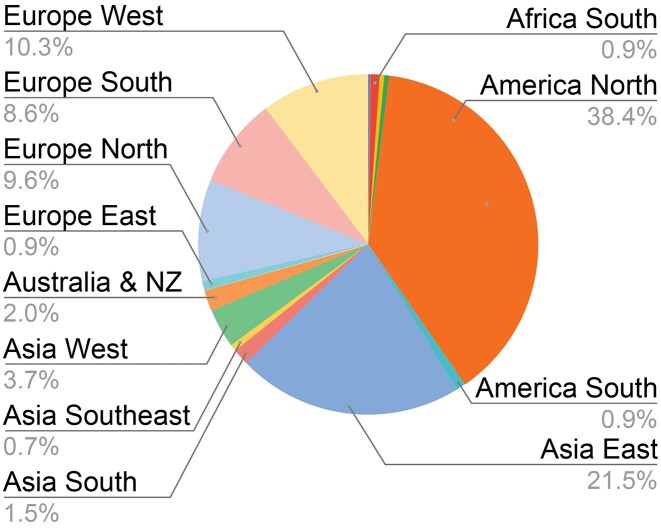
The global distribution of biomedical resources using the DO grouped by sub-continent. Total subcontinents = 15; total countries = 43; total biomedical resources = 376 (July 2023).

Resource developers often grapple with how to measure the success of their projects. In the early days, success was defined by the DO being used by any group outside of our own team. Today, the DO project measures success through a number of metrics (https://disease-ontology.org/about/statistics), including the number of resources citing the DO project publications, the number of resources not just citing but utilizing the DO in their work, and the number of projects that do not cite but do use the DO for their work. Converting to a semi-automated usage workflow identified a 3-fold increase in the number of identified use cases and has greatly reduced the time needed for manual review of publications to identify use cases. Review of new uses in the past two years has offered the project novel insights that suggest new research directions and reveal a clearer picture of the DO’s use and scientific impact. The addition of an assessment of the geographic distribution of use cases revealed a broad global distribution, as measured by where these resources have been developed, across Asia, Africa, Europe, North, Central and South America (Figure 6). This work has identified that the DO has been incorporated into at least 376 biomedical resources developed in 43 countries, with 107 (28.5%) of those resources added since our last NARdb publication.

The increasing number of publications that cite the DO each year demonstrates the utility of the DO (Figure. 5, section 3). As of July 2023, the DO has been cited in over 1832 publications (https://disease-ontology.org/community/publications), 386 (21%) in just the last two years (see https://www.lens.org/lens/search/scholar/list?collectionId=200976). While the DO continues to be used heavily in genomics analysis and data harmonization, it is increasingly used in AI/ML approaches, incorporated into predictive disease models, used in natural language processing and utilized in data preparation to improve model performance. The areas of research using DO have expanded to include data visualization, environmental exposure, drug and natural product use and repurposing, non-coding RNAs, proteomics, and glycoscience.

Expanded usage of the DO is also evident in the increased traffic to disease-ontology.org website as reported by Google Analytics. Since the initial measurement of traffic began in mid-2011, there have been 229 330 unique users to disease-ontology.org with an increase of 69 070 (30%) since our last publication in NARdb. The DO website has been used in 201 countries representing all of the United Nations M49 statistical subregions of the world except Antarctica (https://unstats.un.org/unsd/classifications/Family/Detail/12). Most of the traffic to the disease-ontology.org site is direct (45.8%) but a significant number of users also come to the site via search engines (24.8%) or referrals from other sites, including other resources using the DO (28.5%). The top sites referring users to disease-ontology.org reflect the strong collaborations that have helped to improve and disseminate the disease information in the DO. These include Wikidata: wikipedia.org/wikidata.org (18K+, 27.8%), Mouse Genome Informatics: informatics.jax.org (4.4K+, 6.6%), and the OBO Foundry: obofoundry.org (1.2K+). Examination of the DO’s site usage also highlights the global expansion of the DO and identifies new potential avenues of collaboration, such as with China's National Genomics Data Center: ngdc.cncb.ac.cn (1.2K+).

### Outreach

The DO project utilizes a multi-pronged approach to promote DO and DO-KB resources. Our team produces and shares quarterly Buzz newsletters (https://disease-ontology.org/community/newsletter) and participates in national and international conferences, e.g. NIH Rare Disease Day, ISB Biocuration, ISMB Bio-ontologies and BOSC workshops and International Conference of Biomedical Ontology (ICBO) annual conference. Slide decks are shared via FigShare (https://figshare.com/projects/Human_Disease_Ontology/124345) and SlideShare (https://disease-ontology.org/outreach/slideshare) and informational videos are made available via DO’s YouTube channel (https://disease-ontology.org/outreach/videos). Recent video additions include ‘What is biocuration?’ and ‘Advanced searches of the DO website using relation axioms’. Throughout 2022 and 2023, the DO team has implemented a strategic effort to empower engagement with a broader audience through weekly posts on X (Twitter) (@diseaseontology) and the launching of a DO public slack channel (May 2023). We add links to join this new slack channel on a regular basis to @diseaseontology.

## Future directions

Now that the DO-KB site has launched, the focus of our work in the next months and year turn towards augmenting the DO-KB tools with additional features and content and expanding our suite of federated queries to additional disease, genomic, and proteomic resources. Supplemental queries will be periodically added to the DO-KB SPARQL Sandbox and additional facets related to environmental drivers will augment the Faceted Search Interface. Specifically, relationship terms from RO and from the Disease Drivers ontology will be added to the search options in a future release. DO content expansion will focus on enhancing the DO’s alignment with other large scale biomedical resources e.g. UniProt and GARD. Work will continue on the integration of the WHO BlueBook cancer re-classifications into the DO, e.g. the in progress sarcoma review. A Spanish translation of the DO web pages has begun in order to enhance site accessibility. In 2024 the DO-KB pages will be augmented with spanish versions of each web page. This work will subsequently involve the Spanish translation of DO disease terms, definitions and synonyms. The addition of an ORCID ID-based attribution page is under development to recognize and celebrate the invaluable contributions made to the DO in the past two decades.

## Data Availability

All data produced for this work is made available under the Creative Commons CC0 1.0 license (https://creativecommons.org). Data files and code are available from the project's GitHub repository ((https://github.com/DiseaseOntology/) and from Zenodo (https://doi.org/10.5281/zenodo.10030384).
